# Effect of chromium content on the corrosion resistance of ferritic stainless steels in sulfuric acid solution

**DOI:** 10.1016/j.heliyon.2018.e00958

**Published:** 2018-11-22

**Authors:** Yang Yu, Sayoko Shironita, Kenichi Souma, Minoru Umeda

**Affiliations:** a Department of Materials Science and Technology, Graduate School of Engineering, Nagaoka University of Technology, 1603-1 Kamitomioka, Nagaoka, Niigata, 940-2188, Japan; b Hitachi Industrial Equipment Systems Co., Ltd., 3 Kanda Neribei, Chiyoda, Tokyo, 101-0022, Japan

**Keywords:** Materials science, Electrochemistry, Metallurgical engineering

## Abstract

Due to recent increases in the price of Ni, steel use is currently undergoing a global shift from austenitic stainless steels to ferritic stainless steels. In this study, the corrosion behavior of four types ferritic stainless steels with different Cr contents was investigated to study the effect of Cr content on the corrosion resistance in a sulfuric acid solution. The polarization curves of the ferritic stainless steel with the highest Cr content indicated the best corrosion resistance. No corrosion was observed for the stainless steel with 24 mass% Cr after a potential sweep based on *ex-situ* SEM images. Corrosion resistivity was improved for high Cr content (>24 mass%) stainless steel because it is considered to form a stable passivation layer.

## 1 Introduction

Stainless steels are classified by the three main types: austenitic, ferritic, and martensitic [1, 2, 3, 4, 5]. Among these stainless steels, the corrosion resistances were superior for the Cr-Ni-type austenitic and Cr-type ferritic stainless steels. These stainless steels have been used as kitchen tools, architectural materials, mechanical and chemical-industrial equipment, fuel cell materials, etc. [6, 7, 8]. However, due to the increase in Ni price because of resource exhaustion, use of stainless steels is currently undergoing a global shift from austenitic to Ni-less ferritic stainless steels [9, 10, 11, 12].

The ferritic stainless steel SUS400 series, especially SUS430 in the 1960s and SUS445 in the 1990s, were developed for high corrosion resistance [13, 14]. SUS430 contains 16–18% Cr and SUS445 contains 22% Cr and 1–2% Mo as the corrosion-resistant elements. The corrosion resistance of SUS445 in an acidic solution is as high as that of SUS316, which is a Ni-containing austenitic stainless steel [15]. However, further improvement of corrosion resistance is needed for the application of these stainless steels in acidic and electrochemical environments, such as fuel cell materials.

To realize corrosion resistance of the stainless steels, the natural oxidation of Cr and Fe on the surface to form passivation films composed of oxides/hydroxides is an important factor [16, 17, 18, 19]. The Cr content significantly affects the corrosion resistance; there are a number of papers concerning the relationship between the Cr content amount and the corrosion resistance of stainless steel [20, 21, 22, 23, 24], although few among them report the use of *ex-situ* SEM observation.

Recently, we found that the ferritic stainless steel can show higher corrosion resistance than SUS316 by increasing the Cr content, without any surface treatment, such as nitriding or coating [25, 26, 27]. Ferritic stainless steel without surface treatment should be studied from the viewpoint of materials cost for application as bipolar plates which constitute one of the key components in fuel cells. Investigation of this high-Cr-containing ferritic stainless steel in greater detail revealed the possibility that materials with high corrosion resistance and appreciable electrical conductivity in even harsher environments than usual can be supplied without any surface treatment [28, 29, 30, 31, 32]. In this study, corrosion evaluation experiments were conducted in an acidic solution using a proprietary high-Cr-content ferritic stainless steel in addition to the three SUS 400 series types with the different Cr contents of commercially-available products. The bulk properties were evaluated by X-ray diffraction (XRD) and glow discharge spectroscopy (GDS), and a surface scientific approach was conducted by *ex-situ* scanning electron microscopy (SEM). Corrosion resistance was highly improved at 24 mass% Cr without Ni, while exhibiting a useful level of electrical conductivity (*e.g.* in fuel cells).

## 2 Experimental

### 2.1 Materials

The four types stainless steels used in this study were SUS410 (*t* = 3.9 mm), SUS430 (*t* = 1.0 mm), SUS445 (*t* = 0.1 mm) and 24 mass% Cr-content stainless steel (24CrSS, *t* = 0.1 mm). The chemical compositions (in mass%) of these four types of stainless steels are shown in Table 1. The SUS445 stainless steel contains small amounts of aluminum (Al), molybdenum (Mo), titanium (Ti) and niobium (Nb) elements compared to the other stainless steels. All the four types of stainless steels contain no Ni and contain different Cr amounts.

**Table 1:** Composition of the SUS410, SUS430, SUS445 and 24CrSS stainless steels (mass%) studied.

Sample	Fe	C	Si	Mn	Cr	Mo	Nb	Ti	Al	Ni
SUS410^a^	Bal.	≤0.15	≤0.50	≤1.0	11.5∼13.0	−	−	−	−	−
SUS430^a^	Bal.	≤0.12	≤0.75	≤1.0	16.0∼18.0	−	−	−	−	−
SUS445^b^	Bal.	0.01	0.18	0.20	22.10	1.20	0.23	0.19	0.09	−
24CrSS^c^	Bal.	0.05	0.15	0.30	24.0	−	−	−	0.15	−

a From The Nilaco Corporation.

b From Nisshin Steel Co., Ltd.

c From Hitachi Metals, Ltd.

### 2.2 Electrochemical measurements

The electrochemical measurements were carried out at room temperature using the Electrochemical Analyzer Model 802B (ALS/[H] CH Instruments). To evaluate the corrosion resistance of the SUS410, SUS430, SUS445 and 24CrSS stainless steels, corrosion behavior was studied using an electrochemical three-electrode glass cell. The working, counter, and reference electrodes were the stainless steel specimen, a platinum coil, and an Ag/Ag_2_SO_4_ electrode, respectively. All measured electrode potentials were converted from Ag/Ag_2_SO_4_ to the SHE in this study. A 0.5 mol dm^-3^ H_2_SO_4_ solution was used as the electrolyte. The stainless steels were washed with acetone and distilled water for 5 min during sonication prior to the linear sweep voltammetry (LSV) measurement. A 30-min Ar gas bubbling was also performed. Next, the cathodic treatment for 1 min was conducted at a potential of -0.47 V vs. SHE, then kept for 5 min at the rest potential in the cell. During this holding time, Ar bubbles nucleated on the surface of the sample to remove generated H_2_ gas. The potential sweep was conducted from the rest potential to 1.1 V vs. SHE at a scan rate of 0.33 mV s^-1^ [15]. After the electrochemical measurement, the samples were carefully removed from the cell and cleaned with ethanol. The corrosion resistance of all the samples was evaluated based on the Japanese Industrial Standards (JIS) G0579: 2007 measurement method [33].

### 2.3 Characterization

To evaluate the depth profiles of the elements on the surface of the four types of stainless steels, glow discharge optical emission spectroscopy (GDS) was carried out using a Horiba GD-Profiler 2 instrument. The measured elements included Fe, C, N, Cr, Al, *etc*. In this study, the Cr contents of the four types of stainless steels is the main data to be analyzed.

The X-ray diffraction (XRD) experiments were performed by an XRD-6100 made by Shimadzu. The measurements were carried out in reflection geometry using Cu Kα radiation (λ = 1.5406 Å) generated at 40 kV and 30 mA; 2θ was scanned from 20° to 110° at a rate of 2°·min^−1^.

Scanning electron microscopy (SEM) (JSM-6060A, JEOL Ltd.) was used to observe the surface morphology of the stainless steels before and after the LSV measurements were stopped at the peak of the polarization curves. Micrographs of were obtained at 15 kV.

Finally, the electrical conductivity of the bipolar plate is very important, so the electrical conductivity of the four types of stainless steels was measured by a Mitsubishi Chemical Loresta HP (MCP-T410) electrometer using a four-point probe resistivity technique. As a standard measurement, four-point probe characterization is used to measure the electrical properties of solids and thin films [24, 34, 35, 36]. The resistivity reported here for each stainless steel of this study is the average data of five measurements in different locations.

## 3 Results and discussion

### 3.1 GDS depth profiles

**Fig. 1:**
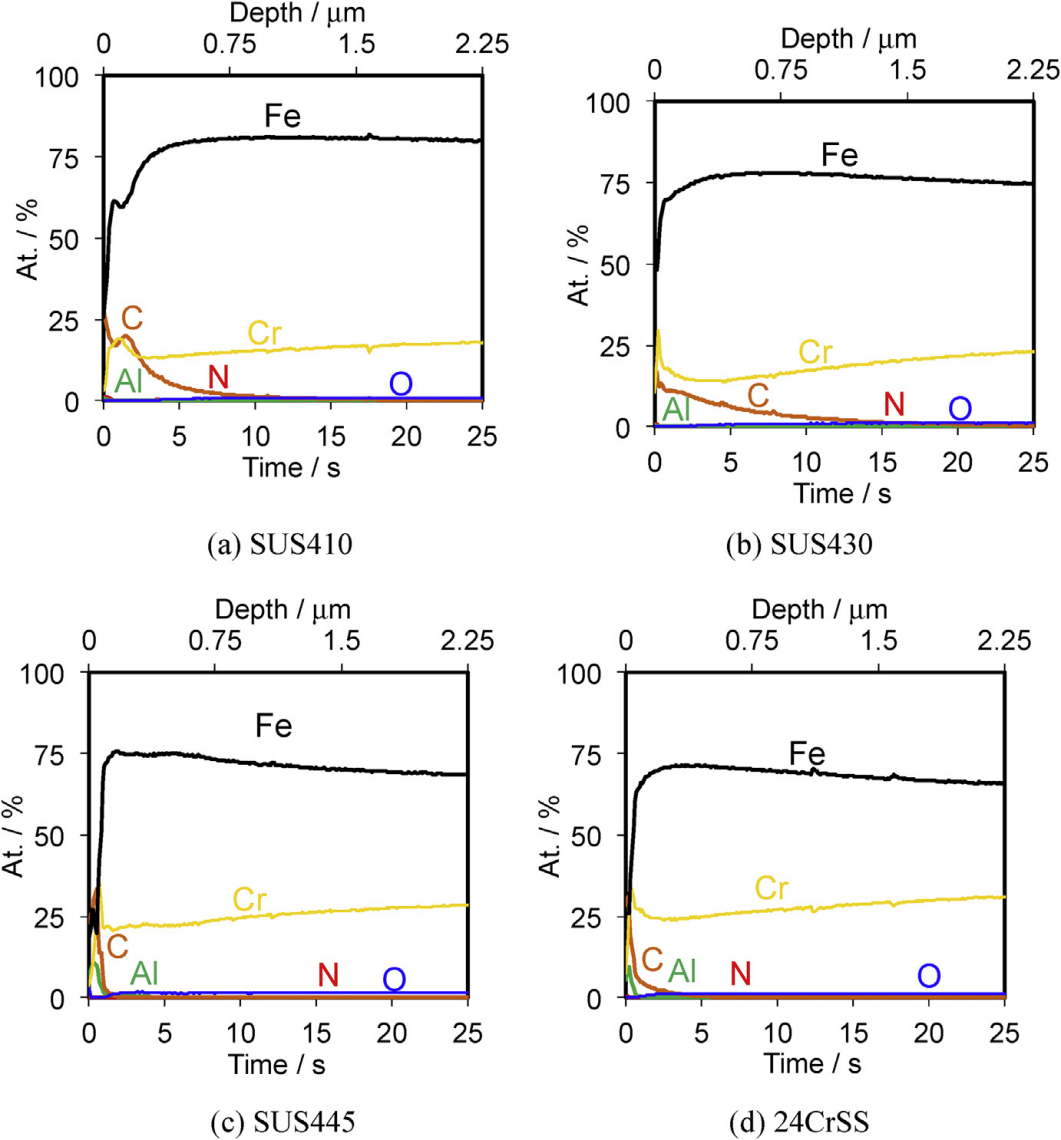
GDS depth profiles of the SUS410, SUS430, SUS445 and 24CrSS stainless steels. (a) SUS410; (b) SUS430; (c) SUS445; (d) 24CrSS stainless steel.

**Fig. 2:**
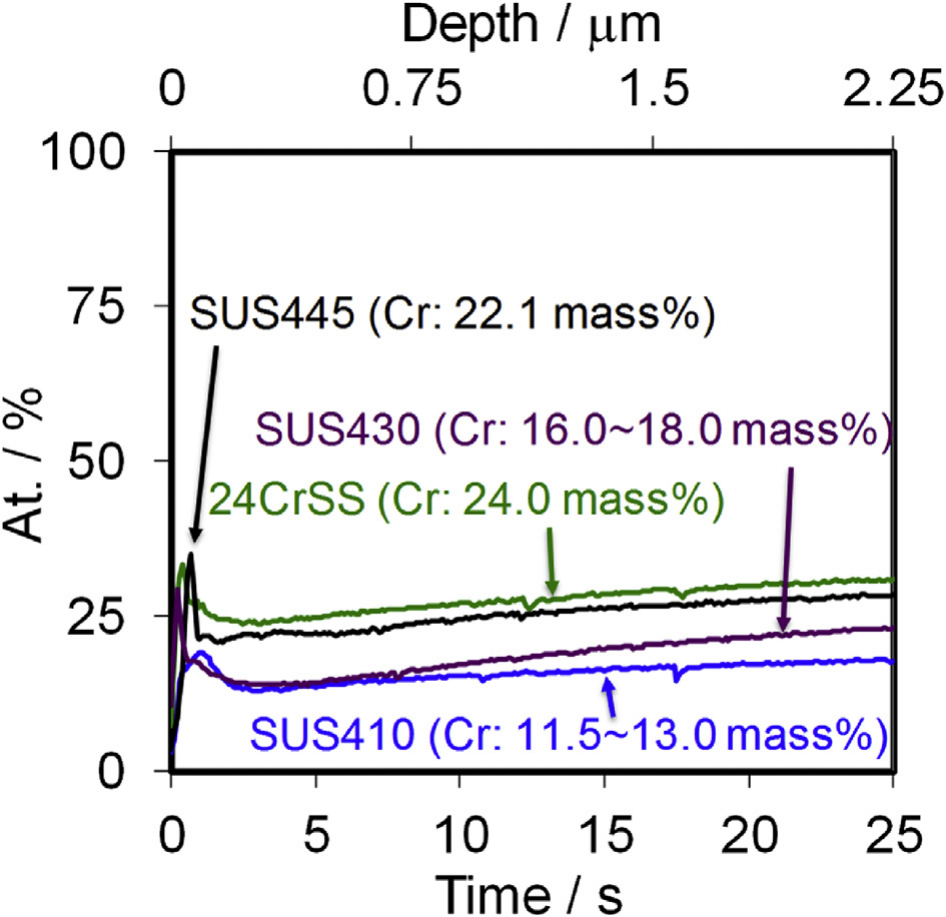
Depth profiles of Cr element of the SUS410, SUS430, SUS445 and 24CrSS stainless steels.

Fig. 1 shows the GDS depth profiles of the SUS410, SUS430, SUS445 and 24CrSS stainless steels. The main compositions of these four types of stainless steels are Fe and Cr; a small amount of C was also detected. The GDS depth profiles of the SUS410, SUS430, SUS445 and 24CrSS stainless steels show different Cr contents in the bulk. The depth profiles of the Cr element of the SUS410, SUS430, SUS445 and 24CrSS samples are shown in Fig. 2. The GDS results show that the Cr contents are increased in the order of SUS410, SUS430, SUS445 and 24CrSS stainless steels. The 24CrSS stainless steel contains the highest content of corrosion-resisting Cr.

### 3.2 X-ray diffraction analysis

**Fig. 3:**
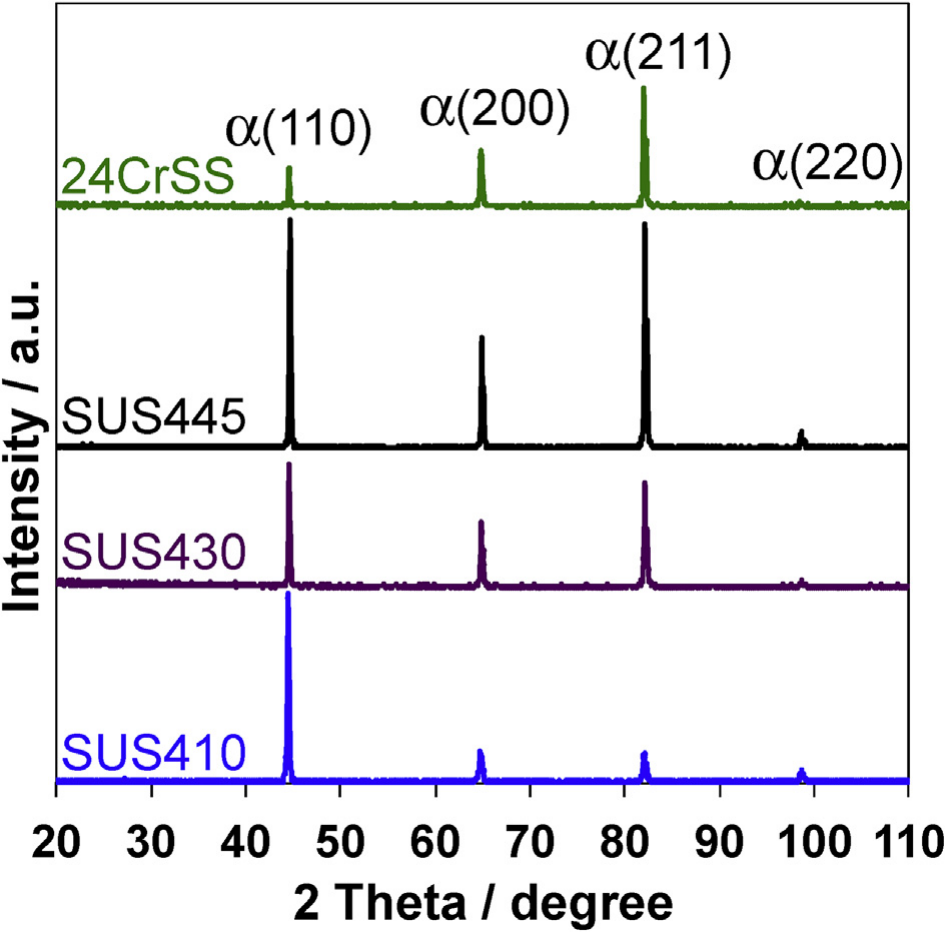
XRD patterns of the SUS410, SUS430, SUS445, and 24CrSS stainless steels.

Fig. 3 shows the XRD patterns of the SUS410, SUS430, SUS445, and 24CrSS stainless steels. The XRD patterns of all the stainless steels show the same diffraction peaks at 44.66°, 64.82°, 82.13° and 98.18°, relative to α-Fe. The peak intensity ratio for (110), (200), (211), and (220) are different at each stainless steel, because of the different rolling directions when they are produced [37]. The XRD results also show that the four types of stainless steels have the ferritic structure, which is a body-centered cubic (BCC) structure.

### 3.3 Corrosion behavior

**Fig. 4:**
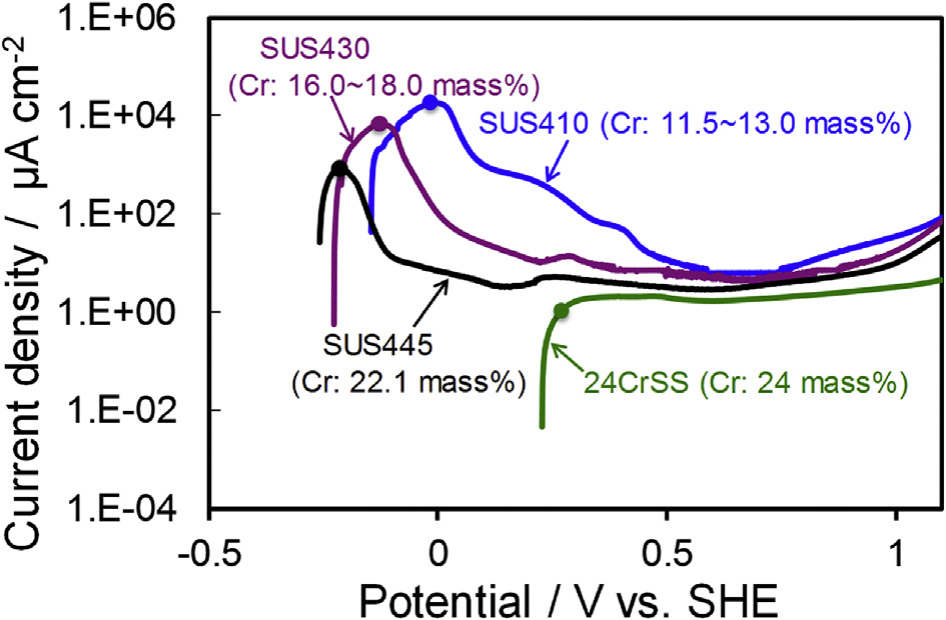
Polarization curves of SUS410, SUS430, SUS445 and 24CrSS stainless steels in Ar-saturated 0.5 mol dm^−3^ H_2_SO_4_ electrolyte. Circle symbols mean the point of SEM observation.

The polarization curves of the SUS410, SUS430, SUS445 and 24CrSS stainless steels in the Ar-saturated 0.5 mol dm^-3^ H_2_SO_4_ electrolyte are shown in Fig. 4. For the SUS410, SUS430 and SUS445 stainless steels, although the polarization characteristics for these three specimens are almost similar in shape, the polarization curve of the SUS445 stainless steel shows the same current density as SUS316 stainless steel in the potential region of 0.25–0.6 V vs. SHE [15]. The formation of passive-current peak shifts due to a negative potential by increasing the Cr content, in increasing order; SUS410, SUS430, and SUS445. Lower current densities are observed at higher Cr-content levels. The SUS410 stainless steel shows the highest current densities due to it having the lowest Cr content.

In the case of the 24CrSS stainless steel, the onset potential shifts toward the positive direction, and there is no active current peak compared to SUS410, SUS430 and SUS445 [24]. This can be explained by the existence of a stable passivation layer even after the cathodic treatment of 24CrSS. The current densities of the 24CrSS sample also decreased indicating a better corrosion resistance of the 24CrSS stainless steel. Because the stainless steel contains a higher Cr content, the best corrosion resistance was observed [27].

### 3.4 Surface morphology

**Fig. 5:**
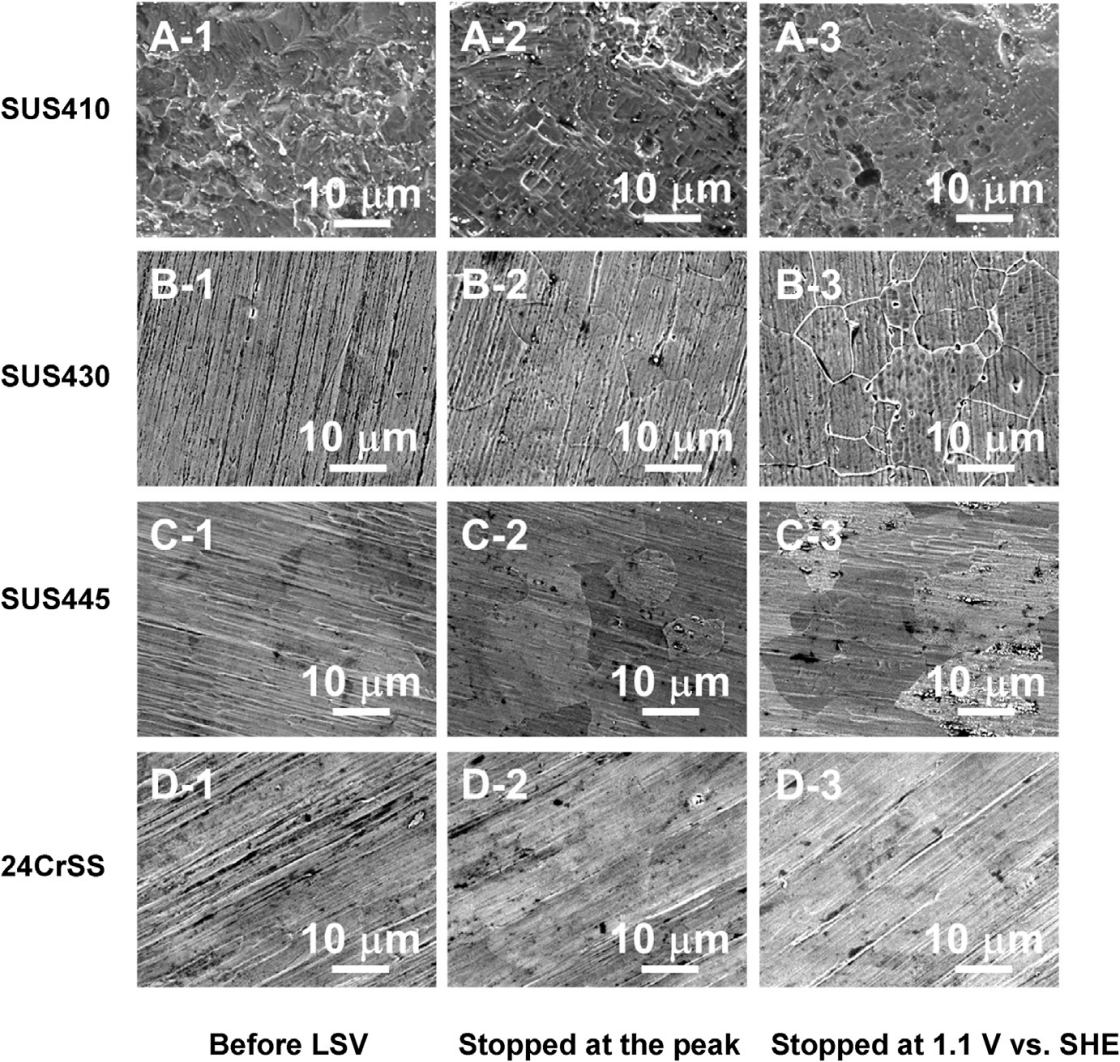
SEM images of (A) SUS410, (B) SUS430, (C) SUS445and (D) 24CrSS stainless steels before/after LSV measurements stopped at the peak of the polarization curves. (A-1, B-1, C-1, and D-1) before LSV. (A-2, B-2, C-2, and D-2) LSV stopped at the point of circle symbols in Fig. 4. (A-3, B-3, C-3, and D-3) after LSV stopped at 1.1 V vs. SHE.

To investigate how the corrosion occurred on the surface of the four types of stainless steels, SEM measurements were carried out to observe the surface morphology of the four types of stainless steels. Fig. 5 shows the SEM images of the four types of stainless steels before and after the LSV measurements stopped at the peak (Fig. 4) and the end of the polarization curves. Compared to before the LSV measurement (Fig. 5(A-1)), the LSV measurement of SUS410 after the rest potential shifts to -0.02 V *vs*. SHE (Fig. 5(A-2)), we can clearly see that the grain of SUS410 was corroded, so the corrosion occurred on the surface of the SUS410 sample during the active region. For the SUS430 stainless steel, compared to before the LSV measurement (Fig. 5(B-1)), the LSV measurement after the rest potential to -0.13 V *vs*. SHE (Fig. 5(B-2)), the surface of the SUS430 stainless steel was corroded during the active region and the grain boundaries became visible due to the chemical attack of the acid solution.

Comparing the SEM images of SUS445 before (Fig. 5(C-1)) and after the LSV measurement from the rest potential to -0.22 V *vs*. SHE (Fig. 5(C-2)), after the LSV measurement, the grain boundaries can still be observed, but not as clearly as that of SUS430 (Fig. 5(B-2)). The surface of the SUS445 stainless steel was also corroded during the active region. The corrosion damage to SUS445 was less than that of the SUS430 stainless steel. At the end of the LSV measurements (Fig. 5(A-3), (B-3), (C-3)), these three stainless steels were significantly corroded. As for the 24CrSS stainless steel, with better corrosion resistance, there was no change to the surface morphology before (Fig. 5(D-1)) and after the LSV measurements (Fig. 5(D-2), (D-3)). No apparent corrosion occurred on the 24CrSS stainless steel. The conjectured reason is that the 24CrSS stainless steel contains the highest content of the corrosion-resisting Cr element, and a passive layer already existed on the surface of the 24CrSS stainless steel before the experiments, thus the polarization curve of the 24CrSS stainless steel has no active region.

### 3.5 Electrical conductivity

It is important for the application of fuel cells to evaluate the materials' electrical conductivity. Therefore, electrical conductivities of SUS410, SUS430, SUS445 and 24CrSS were measured using a Mitsubishi Chemical “Loresta HP” (MCP-T410) electrometer by the four-point probe resistivity technique, and the results are listed in Table 2. The volume resistivity of the four types of stainless steels decreased one order of magnitude compared to that of the graphite carbon. The 0.1 mm-thick 24CrSS stainless steel (with a high corrosion resistance) showed the same electrical conductivity as that of the 0.1 mm thick SUS445 stainless steel. Therefore, we conjecture that it can be used as a bipolar plate, replacing the current graphite carbon.

**Table 2:** Electrical conductivity of the four types of stainless steels, as well as graphite carbon.

Sample name	Thickness (t/mm)	Surface Resistivity (Rs/Ω·□^−1^)	Volume Resistivity (Rs×t: R/Ω·mm^−1^)
SUS410	3.9	2.97E-04	1.16E-03
SUS430	1	2.32E-03	2.32E-03
SUS445	0.1	3.17E-02	3.17E-03
24CrSS	0.1	3.61E-02	3.61E-03
Graphite carbon (Bipolar plate of JARI standard cell)	18	6.29E-04	1.13E-02

## 4 Conclusions

In this study, the corrosion behavior of the four types of ferritic stainless steels with different Cr contents have been experimentally investigated to study the effect of the Cr content on the corrosion resistance of ferritic stainless steels in a sulfuric acid environment. The results revealed the following:

(i) The ferritic stainless steel containing a higher amount of Cr (24 mass%) showed the best corrosion resistance based on its stable passivation layer.
(ii) Based on the SEM images, no corrosion occurred on the 24CrSS stainless steel after the LSV measurement from the rest potential to 0.26 V *vs.* SHE due to its having the highest Cr content of the stainless steels tested in the sulfuric acid solution.

## Declarations

### Author contribution statement

Yang Yu: Performed the experiments; Analyzed and interpreted the data; Wrote the paper.

Sayoko Shironita: Analyzed and interpreted the data; Wrote the paper.

Kenichi Souma: Analyzed and interpreted the data; Contributed reagents, materials, analysis tools or data.

Minoru Umeda: Conceived and designed the experiments; Analyzed and interpreted the data.

### Funding statement

This work was supported by the Cross-ministerial Strategic Innovation Promotion Program (SIP), Cabinet Office, Government of Japan. This work was supported by JSPS KAKENHI Grant Number JP16K06770 and JP18K19127.

### Competing interest statement

The authors declare no conflict of interest.

### Additional information

No additional information is available for this paper.

## References

[1] Suzuki S. (2011). Fundamentals for better use of stainless steels -development history, characteristics and resistance to corrosion- V: indication of selection for application of stainless steels material. J. Soc. Mater. Sci. Jpn.

[2] Gardner L. (2005). The use of stainless steel in structures. Prog. Struct. Eng. Mater..

[3] Sourmail T. (2001). Precipitation in creep resistant austenitic stainless steels. Mater. Sci. Technol..

[4] Xiang-mi Y., Zhou-hua J., Hua-bing L. (2007). Ultra-pure ferritic stainless steels-grade, refining operation, and application. J. Iron Steel Res. Int..

[5] Gelles D.S. (1996). Development of martensitic steels for high neutron damage applications. J. Nucl. Mater..

[6] Zhou F., Li L. (2016). Experimental study on hysteretic behavior of structural stainless steels under cyclic loading. J. Constr. Steel Res..

[7] Baddoo N., Francis P. (2014). Development of design rules in the AISC Design Guide for structural stainless steel. Thin Walled Struct..

[8] Shaigan N., Qu W., Ivey D.G., Chen W. (2010). A review of recent progress in coatings, surface modifications and alloy developments for solid oxide fuel cell ferritic stainless steel interconnects. J. Power Sources.

[9] Mola J., Ullrich C., Kuang B., Rahimi R., Huang Q., Rafaja D., Ritzenhoff R. (2017). Austenitic nickel- and manganese-free Fe-15Cr-1Mo-0.4N-0.3C steel: tensile behavior and deformation-induced processes between 298 K and 503 K (25 C and 230 C). Metall. Mater. Trans. A.

[10] Hastuty S., Nishikata A., Tsuru T. (2010). Pitting corrosion of Type 430 stainless steel under chloride solution droplet. Corrosion Sci..

[11] Yang K., Ren Y. (2010). Nickel-free austenitic stainless steels for medical application. Sci. Technol. Adv. Mater..

[12] Wang H., Brady M.P., More K.L., Meyer H.M., Turner J.A. (2004). Thermally nitrided stainless steels for polymer electrolyte membrane fuel cell bipolar plates Part 2: beneficial modification of passive layer on AISI446. J. Power Sources.

[13] Osozawa K. (2011). I: history of stainless steel and its production. J. Soc. Mater. Sci Jpn..

[14] Khorrami M.S., Mostafaei M.A., Pouraliakber H., Kokabi A.H. (2014). Study on microstructure and mechanical characteristics of low-carbon steel and ferritic stainless steel joints. Mater. Sci. Eng. A.

[15] Yu Y., Shironita S., Nakatsuyama K., Souma K., Umeda M. (2016). Influence of nitriding surface treatment on corrosion characteristics of Ni-free SUS445 stainless steel. Electrochemistry.

[16] Gooch T.G. (1996). Corrosion Behavior of Welded Stainless Steel.

[17] Potgieter J.H., Skinner W., Heyns A.M. (1993). The nature of the passive film on cathodically modified stainless steels. J. Appl. Electrochem..

[18] Hashimoto K., Asami K., Kawashima A., Habazaki H., Akiyama E. (2007). The role of corrosion-resistant alloying elements in passivity. Corrosion Sci..

[19] Luo H., Su H., Dong C., Li X. (2017). Passivation and electrochemical behavior of 316L stainless steel in chlorinated simulated concrete pore solution. Appl. Surf. Sci..

[20] El-Basiouny M.S., Haruyama S. (1976). The polarization behavior of Fe-Cr alloys in acidic sulphate solutions in the active region. Corrosion Sci..

[21] Asami K., Hashimoto K., Shimodaira S. (1978). An XPS study of the passivity of a series of iron-chromium alloys in sulphuric acid. Corrosion Sci..

[22] Dobbelaar J.A.L., Herman E.C.M., De Wit J.H.W. (1992). The corrosion behavior of iron-chromium alloys in 0.5 M sulphuric acid. Corrosion Sci..

[23] Hamm D., Olsson C.-O.A., Landolt D. (2002). Effect of chromium content and sweep rate on passive film growth on iron-chromium alloys studied by EQCM and XPS. Corrosion Sci..

[24] Wang H., Turner J.A. (2004). Ferric stainless steels as bipolar plate material for polymer electrolyte membrane fuel cells. J. Power Sources.

[25] Li C.X., Bell T. (2004). Corrosion properties of active screen plasma nitrided 316 austenitic stainless steel. Corrosion Sci..

[26] Yu Y., Shironita S., Nakatsuyama K., Souma K., Umeda M. (2016). Surface composition effect of nitriding Ni-free stainless steel as bipolar plate of polymer electrolyte fuel cell. Appl. Surf. Sci..

[27] Yu Y., Shironita S., Mizukami T., Nakatsuyama K., Souma K., Umeda M. (2017). Corrosion-resistant characteristics of nitrided Ni-free stainless steel for bipolar plate of polymer electrolyte fuel cell. Int. J. Hydrogen Energy.

[28] Pourbaix M. (1974). Atlas of Electrochemical Equilibria in Aqueous Solutions.

[29] Sasaki K., Shao M., Adzic R., Büchi F.N., Inaba M., Schmidt T.J. (2009). Dissolution and stabilization of platinum in oxygen cathodes. Polymer Electrolyte Fuel Cell Durability.

[30] Wang Z.-B., Zuo P.-J., Chu Y.-Y., Shao Y.-Y., Yin G.-P. (2009). Durability studies on performance degradation of Pt/catalysts of proton exchange membrane fuel cell. Int. J. Hydrogen Energy.

[31] Inoue M., Nakazawa A., Umeda M. (2012). Effect of H_2_O_2_ on Pt electrode dissolution in H_2_SO_4_ solution based on electrochemical quartz crystal microbalance study. Int. J. Hydrogen Energy.

[32] Itaya H., Shironita S., Nakazawa A., Inoue M., Umeda M. (2016). Electrochemical quartz crystal microbalance study of high-rate Pt dissolution in H_2_O_2_-containing H_2_SO_4_ solution with Fe^2+^ ion. Int. J. Hydrogen Energy.

[33] JIS (Japanese Industrial Standards).2007G0579.

[34] Pouraliakbar H., Jandaghi M.R., Khalaj G. (2017). Constrained groove pressing and subsequent annealing of Al-Mn-Si alloy: microstructure evolutions, crystallographic transformations, mechanical properties, electrical conductivity and corrosion resistance. Mater. Des..

[35] Wang T., Ma H.-P., Yang J.-G., Zhu J.-T., Zhang H., Feng J., Ding S.-J., Lu H.-L., Zhang D.W. (2018). Investigation of the optical and electrical properties of ZnO/Cu/ZnO multilayers grown by atomic layer deposition. J. Alloy. Comp..

[36] Li J.C., Wang Y., Ba D.C. (2012). Characterization of semiconductor surface conductivity by using microscopic four-point probe technique. Phys. Proc..

[37] Jandaghi M.R., Pouraliakbar H., Khalaj G., Khalaj M.-J., Heidarzadeh A. (2016). Study on the post-rolling direction of severely plastic deformed Aluminum-Manganese-Silicon alloy. Arch. Civil Mech. Eng..

